# Diagnostic values of peripheral blood T-cell spot of tuberculosis assay (T-SPOT.TB) and magnetic resonance imaging for osteoarticular tuberculosis: a case-control study

**DOI:** 10.18632/aging.202720

**Published:** 2021-03-19

**Authors:** Xiaoliang Li, Junli Wang, Zhigang Yang, Qiongzhu Song

**Affiliations:** 1 Department of Respiratory and Critical Care Medicine, Henan Provincial People’s Hospital, Zhengzhou University People’s Hospital, Zhengzhou, Henan, China; 2 Department of Cardiopulmonary Function, Fuwai Central China Cardiovascular Hospital, Zhengzhou, Henan, China

**Keywords:** osteoarticular tuberculosis, peripheral blood T-cell spot of tuberculosis assay, magnetic resonance imaging, diagnostic accuracy

## Abstract

Objective: Early diagnosis of osteoarticular tuberculosis helps improve patients’ outcomes, but little is known about the accuracy of noninvasive diagnostic methods. This case-control study aimed to assess the diagnostic value of peripheral blood T-cell spot of tuberculosis assay (T-SPOT.TB) and magnetic resonance imaging (MRI).

Methods: Patients suspected with osteoarticular tuberculosis were retrospectively included and diagnosed according to the composite reference standard. T-SPOT.TB was used to detect the number of cells secreting Interferon gamma. Diagnostic performance of T-SPOT.TB and MRI alone and combined were evaluated.

Results: Among the suspected patients, 92 had osteoarticular tuberculosis and 137 non- osteoarticular tuberculosis. T-SPOT.TB assay alone had a higher sensitivity (0.73 vs. 0.60) but a lower specificity (0.69 vs. 0.91 P>0.05) in diagnosing osteoarticular tuberculosis. Combined serial test showed a sensitivity and specificity 0.47, 0.97, respectively, whereas combined parallel test showed a sensitivity and specificity of 0.86, 0.65, respectively. Specificity was higher in the combined serial test than in the T-SPOT.TB assay (P=0.007) or MRI alone (P < 0.001). Furthermore, sensitivity was higher in the combined parallel test than in the T-SPOT.TB assay (P < 0.001) or MRI alone (P < 0.001).

Conclusions: Combined blood T-cell spot of tuberculosis assay and osteoarticular MRI have higher sensitivity and specificity for noninvasive osteoarticular tuberculosis diagnosis, compared with either method alone.

## INTRODUCTION

Tuberculosis (TB) is one of the most serious public health problems worldwide. According to the 2017 World Health Organization statistics, there were about 10 million new TB cases globally and 889,000 new TB cases in China, which accounted for 9% of the global new cases [1]. In fact, China is currently the second largest TB burden country in the world. Because the number of immunodeficient hosts and multidrug-resistant strains increased, the number of atypical OTB cases has increased in recent years. Osteoarticular tuberculosis (OTB) is a common type of TB which accounts for about 3-5% of the total TB incidence and 35-50% of extrapulmonary TB incidence in the world [2]. OTB is defined as the disease caused by *Mycobacterium tuberculosis* infecting bone, joint, synovium and spine. Symptoms onset are usually occult in OTB, and disease progression is slow. The duration of symptoms ranges from several weeks to years. Back pain is the most common symptom, followed by tenderness of the osteoarticular. If the tuberculous lesions were well controlled, the function would not be affected. Missed diagnosis or misdiagnosis, led to serious consequences such as bone joint deformity and paraplegia.

Interferon gamma release assays (IGRAs), which uses enzyme-linked immunospot assay (ELISPOT assay) to quantitatively detect the level of interferon released by the peripheral blood mononuclear cells stimulated by *Mycobacterium tuberculosis* (Mtb) specific antigen, are new techniques widely used in the diagnosis of TB, T cells are sensitized to *Mycobacterium tuberculosis* antigens and the activated effector T cells, both CD4+ and CD8+, produce the cytokine interferon gamma (IFN-γ) when stimulated by these antigens. ELISPOT assay is used to detect the number of cells secreting specific antibodies. Early secretory antigen target 6000 protein (ESAT-6, antigen A) and culture filtrate protein-10 (CFP-10, antigen B) come from a strain specific gene fragment in *Mycobacterium tuberculosis* gene, called region of difference 1 (RD1). This gene fragment is not found in most nontuberculous mycobacteria and all Mycobacterium bovis (including Bacillus Calmette Guerin), so it is much specific as an antigen. T-SPOT.TB is one of the common IGRAs used in the clinical setting. By detecting the number of effector T cells, that is, the number of spot forming cells (SFCS), we can judge whether there is tuberculosis infection. T-SPOT.TB is much more specific than PPD and only takes two days to complete the test [3]. Furthermore, the immune response caused by two antigens is different, which has good complementarity and can improve the positive rate. As a high TB burden country in the world, up to 18.8% of the Chinese population suffers from latent TB infections [4], but these are often undetectable by the T-SPOT.TB assay due to its poor specificity and inability to distinguish between active and latent TB infections. In addition, T- SPOT.TB still has a positive rate of 50% after one year regular treatment, which also affects T- SPOT.TB specificity [5].

MRI is the primary tool of OTB diagnosis. Intervertebral disc destruction, one of the common types of spinal tuberculosis, often manifested as narrowing or disappearing intervertebral disc and showed decreased intensity on T1 weighted images (T1WI) and increased intensity on T2 weighted images (T2WI). Bone collapse, the most common form of spinal tuberculosis, shows decreased intensity on T1WI and increased intensity on T2WI. A mixed signal intensity is also seen in some cases. MRI features of joint tuberculosis include synovitis, synovial fluid, bone collapse, articular cartilage destruction, soft tissue swelling around the joint and cold abscess. Articular cartilage damage is usually characterized by rough and irregular articular surface, local thinning and defect. In later stages, very high signal intensity on T2WI and low on T1WI centrally can reflect an intraosseous abscess in OTB. These findings are usually not obvious during early OTB and there are limitations in the differential diagnosis between TB and other non-TB osteoarthropathy, because of atypical imaging manifestations, whose proportion is about 10-25% [6, 7].

Therefore, a noninvasive, simple, and accurate diagnostic method is urgently needed for osteoarticular TB. In this study, we found that the combination of T- SPOT.TB and MRI may have improved sensitivity and specificity in OTB diagnosis.

## RESULTS

### Baseline characteristics

**Figure 1:**
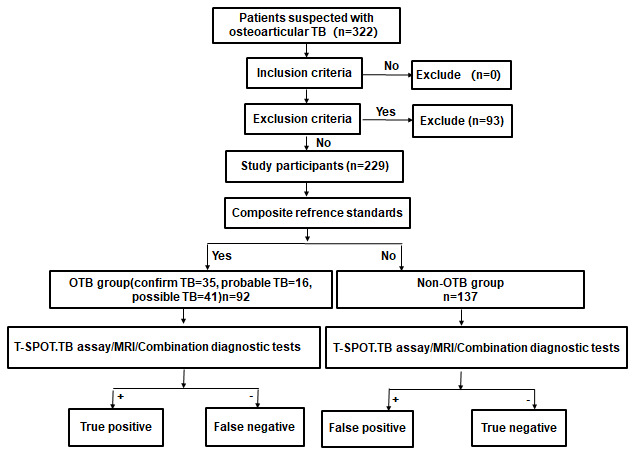
**Study flowchart.**

A total of 322 patients suspected with OTB were identified. After excluding 93 without T-SPOT.TB or MRI or undefined diagnosis or lost to follow up. 229 patients were included in our analyses (Figure 1). Fifty-one patients with OTB had biopsy or surgical specimens available for histopathological examination. 44 (86.3%) patients were detected with granulomatous inflammation and/or caseating necrosis. Positive of Acid-fast bacilli (AFB) were observed in 15 (29.4%) patients, 24 (47.1%) patients were TB-DNA positive in the lesion.

The baseline characteristics of the two groups were similar including age, gender, hypertension, diabetes, and coronary heart disease (Table 1). The proportion of patients with hypoproteinemia, tuberculosis in other organs and history of tuberculosis were significantly higher in the OTB group than the non-OTB group (P < 0.001, P< 0.001 and 0.033, respectively).

**Table 1:** Clinical characteristics of 229 patients with osteoarticular tuberculosis.

**Characteristics**	**OTB group (n = 92)**	**Non-OTB group (n = 137)**	**P value**
Age, y (mean ± SD)	53 ± 17	55 ± 18	0.354
Gender (n, %)			
Male	54 (58.7)	85 (61.6)	0.660
Female	38 (41.3)	52 (38.4)	
Underlying condition or illness (n, %)			
Smoking	30 (32.6)	40 (29.2)	0.583
Hypertension	19 (20.7)	28 (20.4)	0.969
Diabetes	9 (9.8)	19 (13.9)	0.355
Coronary heart disease	3 (3.3)	12 (8.8)	0.099
Arrhythmia	2 (2.2)	5 (3.6)	0.705
Hyperlipidemia	2 (2.2)	6 (4.4)	0.480
Chronic gastritis	2 (2.2)	0 (0)	0.160
Cerebral vascular disease	5 (5.4)	9 (6.6)	0.725
Bronchial asthma	1 (1.1)	2 (1.5)	1.000
Chronic obstructive pulmonary disease	1 (1.1)	5 (3.6)	0.406
Rheumatoid arthritis	0 (0)	3 (2.2)	0.276
Ankylosing spondylitis	3 (3.3)	4 (2.9)	1.000
Hypoproteinemia	73 (79.3)	78 (56.9)	< 0.001
Previous TB infection history	12 (13.0)	7 (5.1)	0.033
Concurrent TB at other sites	13 (14.1)	0 (0)	< 0.001

The classifications of the 137 non-OTB patients are summarized in Table 2. Among the 137 patients, 56 had purulent spondylitis, 22 had osteoarticular metastasis tumor, and 15 had brucella spondylitis.

**Table 2:** Classification of 137 non- osteoarticular tuberculosis patients.

**Classification of diseases**	**Proportion**
Purulent spondylitis	56 (40.9%)
Osteoarticular metastasis tumor	22 (16.1%)
Brucella spondylitis	15 (10.9%)
Disc herniation	16 (11.7%)
Osteoarticular tumor	13 (9.5%)
Synovitis	5 (3.6%)
Bone fracture	4 (2.9%)
Multiple myeloma	3 (2.2%)
Osteoarthritis	1 (0.7%)
Osteonecrosis of the femoral head	1 (0.7%)
Langerhans cell histiocytosis	1 (0.7%)

### Representative examples of T-SPOT.TB and MRI

**Figure 2:**
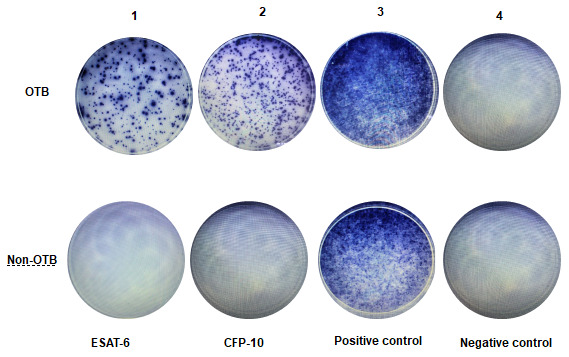
**Representative examples of T-SPOT.TB assay in non-OTB and OTB patients.** This figure shows representative results of the T-SPOT.TB assay from two patients. Figure 2-OTB shows a positive result from an OTB patient. Figure 2-Non-OTB shows a negative result from a non-OTB patient.

A representative example of the T-SPOT.TB results was shown in Figure 2. In OTB group, the first panel shows the result of antigen A, in which 155 SFCs/2.5×10^6^ PBMCs were stimulated by ESAT6 peptides. The second panel shows the result of antigen B, in which 200 SFCs/2.5×10^6^ PBMCs were stimulated by CFP10 peptides The third panel shows the result of positive control, in which PBMCs were stimulated by phytohemagglutinin, The fourth panel shows the result of negative control, in which 2.5 x 10^6^ PBMCs were unstimulated. Non-OTB shown negative results of OTB, in which 2.5 x 10^6^ PBMCs were unstimulated by ESAT6 and CFP10 peptides.

**Figure 3:**
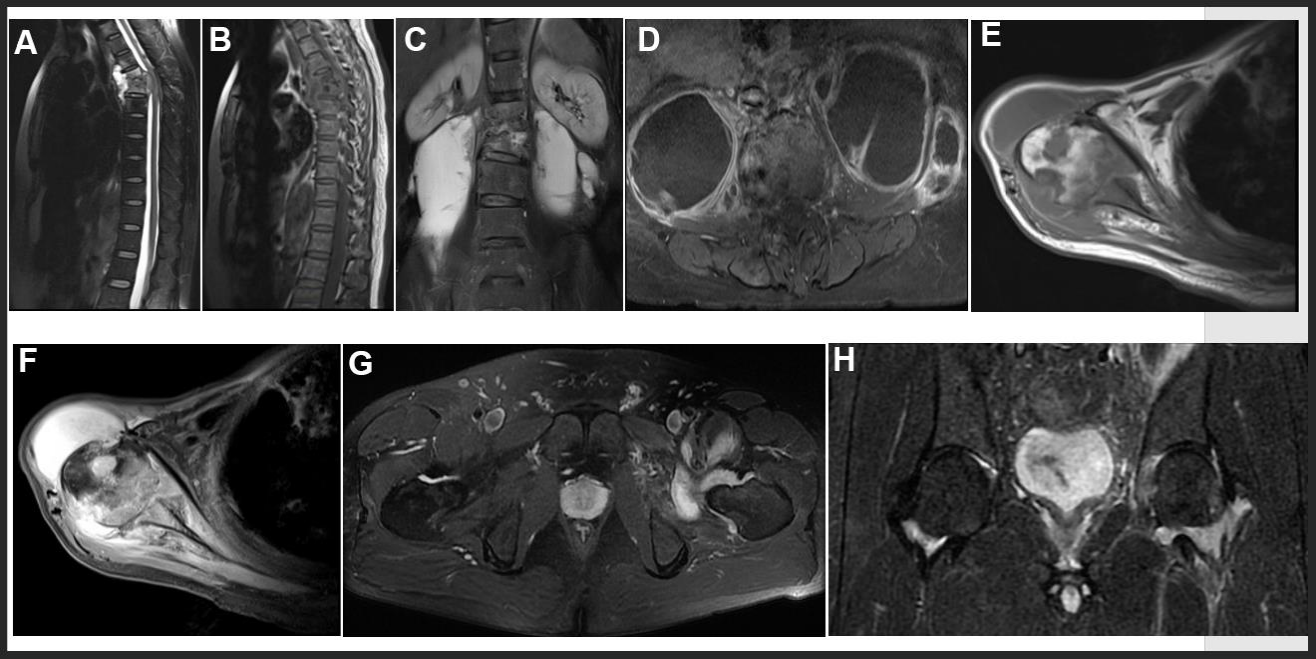
**Representative examples of MRI in OTB patients.** (**A**, **B**) Thoracic spine tuberculosis: showing destruction of vertebral and paravertebral abscess. (**C**, **D**) Lumbar spine tuberculosis: showing destruction of vertebral and psoas abscess. (**E**, **F**) Right shoulder tuberculosis: showing worm like destruction of the right humeral head with effusion in the articular cavity. (**G**, **H**) Left hip joint tuberculosis: showing left hip joint synovitis and synovial fluid.

MRI features in our study included intervertebral disc destruction (Figure 3A, 3B), para- or intra-osseous abscess (Figure 3A, 3B), subligamentous spread, psoas abscess (Figure 3C, 3D), worm like destruction (Figure 3E, 3F), and synovitis and synovial fluid (Figure 3G, 3H).

### Diagnostic value of T-SPOT.TB, MRI

The sensitivity and specificity of T-SPOT.TB and MRI in OTB diagnosis were 0.73 vs 0.60 and 0.69 vs 0.91, respectively (Table 3). 92 OTB patients were divided into four groups (< 6, 6-12, 12-24, > 24months) according to the duration of symptoms before hospitalization [8]. The positivity of T-SPOT.TB in the four groups were 69.4% (43/62), 84.6% (11/13), 100% (5/5) and 66.7% (8/12), respectively. In comparison, the positivity of MRI in the four groups were 59.7% (37/62), 76.9% (10/13),60% (3/5) and 41.7% (5/12), respectively. However, there was no difference in T-SPOT.TB and MRI positivity among these groups.

**Table 3:** Comparison of various diagnostic parameters of T-SPOT.TB, MRI, and combination tests (n = 229).

	**Sensitivity (%)**	**Specificity (%)**	**PPV**	**NPV**	**LR+**	**LR-**	**Accuracy (%)**
T-SPOT.TB	0.73	0.69	0.61	0.79	2.35	0.39	0.71
MRI	0.60	0.91	0.82	0.77	6.83	0.44	0.79
Serial test	0.47	0.97^#^	0.91	0.73	16.01	0.55	0.77
Parallel test	0.86*	0.65	0.62	0.87	2.45	0.22	0.73

PPV, positive predictive value; NPV, negative predictive value; LR+, positive likelihood ratio; LR-, negative likelihood ratio; ^#^Comparison of the specificity between T-SPOT.TB and Serial test, P=0.007, comparison of the specificity between MRI and Serial test, P<0.001; *Comparison of the sensitivity between T- SPOT.TB and Parallel test, P<0.001, comparison of the sensitivity among MRI and Parallel test, P<0.001.

The combination test of T-SPOT.TB and MRI was performed as either serial test or parallel test. Serial test referred to the sequential performance of T-SPOT.TB and MRI. Diagnosis was established when the results of both tests were positive. The sensitivity and specificity of the serial test in OTB diagnosis were 0.47, 0.97, respectively. Parallel test meant that both screening tests were performed simultaneously and diagnosis was established as long as one of the results was positive. The sensitivity and specificity of the parallel test were 0.86, 0.65, respectively. Specificity was higher in the combined serial test than in the T-SPOT.TB assay (P=0.007) or MRI alone (P < 0.001). Furthermore, sensitivity was higher in the combined parallel test than in the T-SPOT.TB assay (P < 0.001) or MRI alone (P < 0.001).

### Correlations among SFCs, positive rate and clinical features

The clinical manifestations of OTB were influenced by various factors, including gender, age, site, underlying illness and other factors. These differences might be reflected by differences in T-SPOT.TB. However, whether the diagnostic accuracy of T-SPOT.TB influenced by clinical features was unknown. The correlations among the number of SFCs, positive rate and various clinical features of the 92 OTB patients are shown in Table 4. The number of SFCs and positive rate were influenced by gender and cumulative spinal segments.

**Table 4:** Factors associated with the number of SFCs and positive rate of T-SPOT.TB test among 92 osteoarticular tuberculosis patients.

		**Number**	**T-SPOT.TB test**					
			**ESAT-6**		**CFP-10**		**Positive rate**	
			**SFC**	**P^1^**	**SFC**	**P^2^**	**Positive rate(%)**	**P^3^**
Gender	Male	54	11	0.021	5	0.021	36(66.7)	0.113
	female	38	39		25		31(81.6)	
Age (y)	<60	56	30	0.997	11	0.775	39(69.6)	0.392
	≥60	36	20		8		28(77.8)	
Underlying disease	Yes	51	16	0.087	6	0.075	36(70.6)	0.590
	No	41	40		22		31(75.6)	
Lesion site	Spine	78	19	0.140	7	0.059	56(71.8)	0.751
	Joint	14	50		60		11(78.6)	
Abscess	Yes	29	38	0.439	22	0.339	29(78.4)	0.424
	No	42	16		7		39(70.9)	
Number of vertebral segments	≤2	64	13	0.045	6	0.047	43(67.2)	0.016
	>2	14	34		23		14(100)	
Previous TB infection history	Yes	12	43	0.557	10	0.775	9(75.0)	1.000
	No	80	20		10		58(72.5)	

## DISCUSSION

### Major findings

This study showed that the sensitivity and specificity of the combination tests were significantly higher than that of the T-SPOT.TB or MRI test alone. Parallel test improved sensitivity while serial test had higher specificity. We found that the positive likelihood ratio (LR+) was substantially increased in the serial test (16.01) compared with T-SPOT.TB alone (2.35), indicating the serial test had a significantly higher diagnostic value for OTB. In contrast, the negative likelihood ratio (LR-) decreased from 0.39 in the T-SPOT.TB test alone to 0.22 in the parallel test, indicating the parallel test was more appropriate for excluding OTB. As soon as OTB is diagnosed, early treatment not only prevents the progression of the disease and reduces the occurrence of deformities, but also preserves osteoarticular function and even cures the disease without surgery [9]. Therefore, combined T-SPOT.TB and MRI parallel test is recommended to reduce the rate of misdiagnosis and improve the sensitivity of OTB diagnosis.

### Dilemmas of noninvasive diagnosis of OTB in current practice

The gold standards for OTB diagnosis are bacteriological and/or histopathological confirmation of Mtb in the synovial fluid or biopsied tissues [10]. Bone tissue biopsy stained for AFB is only 20-40% accurate in the identification of active OTB [11]. On the other hand, conventional Mtb culture techniques are time-consuming (usually 6-8 weeks) and have a low positive rate [8]. In addition, since the bacterial burden is low at the lesion site, it is sometimes difficult to puncture into the core of the lesion, which in turn limits the clinical application of CT-guided aspiration or surgical resection of the lesion [12, 13]. Therefore, non-invasive, accurate, and simple diagnostic tests are urgently needed for OTB. Retrospective study showed the sensitivity and specificity of the T-SPOT.TB and MRI. We combined T-SPOT.TB and MRI for diagnosis of osteoarticular tuberculosis, and found that the combined method had the highest diagnostic value compared with T-SPOT.TB or MRI alone. This method also took the advantages of being noninvasive, accurate and simple.

### T-SPOT.TB for OTB diagnosis

T-SPOT.TB has been widely used for the diagnosis of extrapulmonary TB in recent years [14]. However, the diagnostic value of T-SPOT.TB for extrapulmonary TB varies with infection sites [15–17]. In particular, the sensitivity and specificity of T-SPOT.TB in OTB diagnosis were reported to vary between 81-93% and 33-88% in different studies, respectively [18–21]. In this study, we found that T-SPOT.TB had a 73% sensitivity, lower than that reported in the literature. Among 31 patients with negative T-SPOT.TB results in OTB group, subgroup analysis revealed that 2 <18 years old patients were negative for the T-SPOT.TB test, which was consistent with previous studies [22, 23]. Besides, negative T-SPOT.TB results were found in 8 patients aged > 60 years old and in 17 patients with hypoproteinemia. Age and malnutrition suppressing the systemic immune response, were associated with the positive rate of T-SPOT.TB in OTB patients. As previous studies showed, aging and malnutrition are risk factors for false-negative results [24, 25]. We also found that the T- SPOT.TB positive rate was higher in patients with joint TB (78.6%, 11/14) than in those with spinal TB (71.8%, 56/78). This difference in positive rate may be due to varying degrees of immune responses to TB in different parts of the body [15, 17]. Furthermore, we found that the T-SPOT.TB positive rate was higher in OTB patients with > 2 cumulative affected spinal segments (100%, 14/14) than in those with ≤ 2 affected vertebral segments (67%, 43/64) (P = 0.016). A possible explanation for this is that a larger OTB lesion is associated with a higher bacterial burden, which induces a greater immune response and hence a higher T-SPOT.TB positive rate.

### MRI for OTB diagnosis

Since MRI has a higher resolution for soft tissue deformations, it can detect abnormalities during the early inflammatory infiltration stage of the disease and thus has early diagnostic value in OTB diagnosis. As a result, MRI should be performed as soon as OTB is suspected [26]. In this study, the sensitivity (60%) of MRI in OTB diagnosis was lower than those reported in previous studies [7, 27]. Further analysis showed that 37 OTB patients were MRI-negative and this was mainly caused by the atypical manifestations of the lesions, which reduced the sensitivity of MRI. Previous studies reported that the proportion of atypical imaging patients in OTB is about 10-25% [4, 6]. Moreover, imaging examination alone is unable to distinguish TB infections from other pathogen infections. Therefore, imaging should be used in conjunction with other tests in the clinical diagnosis of patients suspected with OTB.

### Combination of T-SPOT.TB and MRI for OTB diagnosis

This study explored combination of T-SPOT.TB and MRI to improve OTB diagnosis during an early disease stage. The combined use of T-SPOT.TB and MRI had a higher sensitivity and specificity than that of T-SPOT.TB and MRI alone for OTB diagnosis. Besides, we also found that T- SPOT.TB and MRI complement each other and improve the diagnostic accuracy. 42 patients in the control group were positive for the T-SPOT.TB test, which may be caused by the presence of latent infections, and this reduced the specificity of T-SPOT.TB. Further analysis showed that only 9.5% (4/42) of patients was MRI-positive, suggesting that the combined use of MRI can improve the accuracy of diagnosis if the patient is not suspected with OTB but is positive for the T- SPOT.TB test. On the other hand, 37 patients in the case group were MRI-negative but 25 of these 37 patients (67.6%) were positive for T-SPOT.TB, suggesting that we should combine T-SPOT.TB with MRI if the patient is suspected with OTB but negative for MRI, especially in patients presenting with atypical MRI features. Therefore, the combination of T-SPOT.TB and MRI is a rapid and highly sensitive method for OTB diagnosis.

### Limitations

There were several limitations in our study. First, we used the composite reference standards as the gold standard for OTB diagnosis. Since China is a high risk country for TB, etiological examination alone is not sufficiently sensitive for the evaluation of new diagnostic tests. Second, this was a retrospective study and its quality may not be as good as a cross-sectional study. Third, because only a limited number of immunocompromised patients were examined in this study, we could not discuss the relationship between sensitivity of the two tests and the immune function of OTB patients. Thus, further studies are needed to assess the diagnostic values of two tests in immunocompromised patients. Fourth, the T-SPOT.TB is unable to differentiate active with latent TB infection. Therefore, a positive T-SPOT.TB assay result might be due to LTBI or a previous history of TB, which could not distinguish active TB from LTBI might limit its application in the detection of active TB, especially in countries with a high prevalence of TB, such as China. In addition, the patients in this study were from a tertiary hospitals in China. These patients had relatively severe clinical features, and therefore might introduce selection bias in the subject population. In the future, cross-sectional studies with a larger sample size will be needed to further confirm the accuracy of combination T-SPOT.TB and MRI tests for OTB diagnosis.

## CONCLUSIONS

In summary, T-SPOT.TB combined with MRI can enhance the diagnostic accuracy of OTB and is a noninvasive, simple and accurate diagnostic method worthy of further clinical study and validation.

## MATERIALS AND METHODS

### Study participants

A total of 322 patients suspected with OTB and hospitalized in Henan Provincial People's Hospital between January 2014 and October 2020 were screened for this case-control study. Patients were included if they met the following criteria: (1) Underwent MRI examination of the lesion site; (2) Underwent peripheral blood T-SPOT.TB testing; (3) Diagnosed with osteoarthropathy during admission; (4) Had clinical data of interest, including age, sex, clinical manifestations, and pathological and microbiological test results. All patients were followed up for at least 2 months.

### Diagnostic criteria

Patients were diagnosed according to the composite reference standards (CRS), which comprised clinical, laboratory, histopathological, and radiological test and follow-up data [28]. Patients were divided into (1) Confirmed OTB: Identification of *Mycobacterium tuberculosis* by smear, culture, Xpert or PCR; (2) Probable OTB: Identification of granulomatous inflammation and/or caseating necrosis in the osteoarticular biopsy by histopathology; Clinical symptoms and imaging features were consistent with OTB; Symptoms and MRI results were improved after 2 or more months of anti-TB treatment; (3) Possible OTB: Lack of bacteriological and pathological evidence, but clinical features were consistent with OTB; Symptoms and MRI results were improved after 2 or more months of anti-TB treatment; and (4) Non-OTB: Diagnosed with other osteoarticular diseases or the clinical symptoms were not consistent with OTB, and the patient recovered without anti-TB therapy (Figure 1).

### Peripheral blood T-SPOT.TB assay

The T-SPOT.TB test was performed according to the manufacturer’s instructions (Shanghai Fosun Medical Technology Development Co., Ltd.). The steps were as follows: (1) A 8ml blood sample was collected from the patient. (2) The blood with an equal volume of Roswell Park Memorial Institute (PRMI) 1640 medium was laid carefully onto Ficoll-Paque Plus and centrifuged at 1000 g for 22 min at room temperature (18-25° C). The white, cloudy band of PBMCs was collected using a pipette and transferred to a 15mL conical centrifuge tube. (3) The volume was brought to cell culture medium. Centrifuged at 600 g for 7 minutes and repeated. (4) The supernatant was poured off and resuspended the pellet in 0.7mL cell culture medium. (5) The 10μL of the final cell suspension was added 40μL 0.4%(w/v) Trypan Blue solution for manual counting with a Neubauer hemocytometer. An appropriate aliquot was placed onto the hemocytometer and counted the cells in the grid. PBMC numbers between 200,000 and 300,000 per well have been shown to give consistent T-SPOT.TB test results. (6) 500μL of the final cell suspension was prepared at a concentration of 2.5x10^5^ cells/100μL. (7) Added in the Plate and the Controls: i. Added 50μL cell culture medium to each negative control well. ii. Added 50μL ESAT-6 (antigen A) solution to each well required. iii. Added 50μL CFP10(antigen B) solution to each well required. iv. Added 50μL phytohemagglutinin solution (positive control) to each cell functionality control well. (8) To each of the 4 wells to be used for a patient sample, added 100μL of the patient’s final cell suspension (containing 250,000 cells). The plate (96 well plate with nitrocellulose membrane at the bottom, precoated with interferon - γ antibody) with the lid was incubated on in a humidified incubator at 37° C with 5% CO_2_ for 16-20 hours. (9) The plate was removed from the incubator and discarded the cell culture medium. 200μL PBS solution was added to each well, discarded the PBS solution, repeated the well washing an additional 3 times. (10) 50μL working strength Conjugate Reagent solution was added to each well and incubated at 2-8° C for 1 hour, discarded the conjugate and performed the four PBS washes as described in steps 9 above. 50μL Substrate Solution was added to each well and incubated at room temperature for 7 minutes. The plate was washed and dried thoroughly. The number of distinct, dark blue spots on the membrane of each well can be counted directly using a magnifying glass.

A response was classified as positive: (1) If The number of spots in control was 0-5, the number of spots for early secretory antigenic target-6 (ESAT-6) or culture filtrate protein-10 (CFP-10)was ≥ 6, after subtracting the number of spots in the negative control well. (2) If The number of spots in control was 6-10, positive response was defined that the number of spots for early secretory antigenic target-6 (ESAT-6) or culture filtrate protein-10 (CFP-10) was at least twice ≥ the control wells.

### Histopathological examination

Biopsy specimens obtained by surgical resection or CT-guided aspiration were fixed in 4% formalin, paraffin embedded, and stained with hematoxylin–eosin or Ziehl–Neelsen dyes. TB-DNA was measured by PCR using the ABI 7500 thermocycler (ABI, USA). Fluid specimens were stained with the Ziehl-Nielson dyes for the detection of acid fast bacilli (AFB).

### Serial and parallel tests of T-SPOT.TB and MRI

The T-SPOT.TB test and MRI results were independently entered into the computer by an imaging technician and a test technician who were blinded to patients’ diagnosis. All bone biopsy specimens were judged by two pathologists independently, and any disagreement in final diagnosis was resolved by a senior physician. The pathologists were blinded to the T- SPOT.TB and MRI results.

The T-SPOT.TB and MRI combination test was performed as a serial test and a parallel test.

### Sample size calculation

Sample size was calculated according to the formula n = [Ua^2^P(1-P)]/δ^2^ [29], where Ua is 1.96 for a 95% confidence level. When used to calculate patients group, P represents sensitivity, and P represents specificity when used to calculate the control group. δ is the allowable error, which is usually set between 0.05 and 0.1. Based on a δ of 0.1, previous studies showed that the sensitivity and specificity of T-SPOT.TB in the diagnosis of OTB are 86.7 and 61.9%, respectively [30], the calculated sample size for this study was 134 patients, including 44 OTB patients and 90 controls. A final total of 322 patients were included in our study, including 92 OTB patients and 137 controls.

### Statistical analysis

Categorical variables were expressed as number (percentage) and compared using the chi-square test (gender, hypoproteinemia, previous TB infection history). Continuous variables were expressed as mean ± standard deviation or median and compared using the independent sample t test or the Mann-Whitney U test (age, SFCs). Diagnostic value (T-SPOT.TB, MRI, serial test and parallel test) was assessed by sensitivity, specificity, LR+, LR-, positive predictive value (PPV), negative predictive value (NPV), and accuracy. A P value<0.05 was considered statistically significant. Statistical analysis was performed using the SPSS software version 23.0 (SPSS Inc., Chicago, IL, USA).
